# A modified cardiac triage strategy reduces door to ECG time in patients with ST elevation myocardial infarction

**DOI:** 10.1038/s41598-021-86013-8

**Published:** 2021-03-18

**Authors:** Hung-Yuan Su, Jen-Long Tsai, Yin-Chou Hsu, Kuo-Hsin Lee, Chao-Sheng Chang, Cheuk-Kwan Sun, Yu-Han Wang, Shu-Ching Chi, Chih-Wei Hsu

**Affiliations:** 1 Department of Emergency Medicine, E-Da Hospital and I-Shou University, No.1, Yida Road, Jiao-su Village, Yan-Chao District, Kaohsiung City, 82445 Taiwan; 2 School of Chinese Medicine for Post Baccalaureate, I-Shou University, Kaohsiung City, Taiwan; 3 School of Medicine for International Students, I-Shou University, Kaohsiung City, Taiwan; 4 Medical Quality Department, E-Da Hospital, Kaohsiung City, Taiwan

**Keywords:** Cardiac device therapy, Health policy

## Abstract

Timely performing electrocardiography (ECG) is crucial for early detection of ST-elevation myocardial infarction (STEMI). For shortening door-to-ECG time, a chief complaint-based “cardiac triage” protocol comprising (1) raising alert among medical staff with bedside triage tags, and (2) immediate bedside ECG after focused history-taking was implemented at the emergency department (ED) in a single tertiary referral center. All patients diagnosed with STEMI visiting the ED between November 2017 and January 2020 were retrospectively reviewed to investigate the effectiveness of strategy before and after implantation. Analysis of a total of 117 ED patients with STEMI (pre-intervention group, n = 57; post-intervention group, n = 60) showed significant overall improvements in median door-to-ECG time from 5 to 4 min (*p* = 0.02), achievement rate of door-to-ECG time < 10 min from 45 to 57% (*p* = 0.01), median door-to-balloon time from 81 to 70 min (*p* < 0.01). Significant trends of increase in achievement rates for door-to-ECG and door-to-balloon times (*p* = 0.032 and *p* = 0.002, respectively) was noted after strategy implementation. The incidences of door-to-ECG time > 10 min for those with initially underestimated disease severity (from 90 to 10%, *p* < 0.01) and walk-in (from 29.2 to 8.8%, *p* = 0.04) were both reduced. In conclusion, a chief complaint-based “cardiac triage” strategy successfully improved the quality of emergency care for STEMI patients through reducing delays in diagnosis and treatment.

## Introduction

Primary percutaneous coronary intervention (PCI) is the gold-standard treatment for patients with ST-elevation myocardial infarction (STEMI)^1,2^.Timely coronary artery reperfusion after arriving at emergency department (ED) is important for reducing mortality and morbidity rates for patients with STEMI^3–5^. Door-to-Balloon Alliance has chosen key strategies for improving door-to-balloon (DTB) time, which included the activation of the catheterization laboratory with a single call by emergency physicians, completion of PCI team preparation within 20–30 min after the call, rapid data feedback, adoption of a team-based approach, and administrative support^6,7^. Additionally, the American College of Cardiology and American Heart Association (ACC/AHA) has also recommended the target times of door-to-ECG (DTE) within 10 min and DTB within 90 min, respectively, which have become the benchmark for the management of acute coronary syndrome worldwide^1^.

Rapid performance of electrocardiography (ECG) for STEMI identification is crucial to achieving coronary artery reperfusion. Some studies have shown that improving DTE time can shorten DTB time^8–10^. Although a previous large-scale multicenter study has demonstrated no significant reduction in 30-day in-hospital mortality rate for STEMI patients achieving the target time of DTB within 90 min, improving DTE and DTB times should still be persistently emphasized owing to the potential benefits of long-term reduction in mortality, improvement in left ventricular function, and decreasing the number of admissions for heart failure^11^. On the other hand, only 20–30 percent of patients with cardiac ischemic symptoms achieved the target time of DTE within 10 min^8,12^ so that several quality improvement programs had been proposed for shortening the DTE time. Among them, triage ECG, which is the acquisition of ECG during triage before history-taking by emergency physicians, is widely implemented in the ED setting^4,10,13–15^. However, the downside of triage ECG is that, in addition to the need for setting up ECG equipment, technicians and space in the triage area, indiscriminate ECG for patients with suspected STEMI is not cost-effective and can be time-consuming so that the care for other patients could be delayed^16^. To address this issue, Coyne et al. created a “cardiac triage” designation to incorporate triage ECG into an improved patient disposition during the triage process, which has been reported to shorten the DTE time^17^. Although this combined approach was successful in reducing DTE time, some concerns, such as the increased workload among medical staff, interruption and distraction at work as well as a high cost of employing specialized personnel with a low yield of STEMI identification, were raised in a few studies^16,18^.

Therefore, our study modified the combined process to evaluate if cardiac triage alone can improve ECG performance in ED by hanging a red warning tag on the bedside of patients who were suspected of having STEMI by the triage nurse and placing their medical charts in a designated area to expedite subsequent managements, including prompt history-taking by emergency physicians and ECG. The current study aimed at assessment of DTE and DTB time and related factors associated with delayed ECG acquisition.

## Methods

### Study design and patient population

The current study was conducted in a 1251-bed tertiary referral center that had 66,000 emergency visits per year. From November 2017 to January 2020, the electronic medical records of all adult patients (≧ 18 years old) who were diagnosed with STEMI in ED and subsequently received primary PCI in the ED were retrospectively reviewed by a STEMI quality control team every month as a standard procedure of a medical quality improvement program of the institute. The team, which comprised cardiologists, emergency physicians, triage nurses, and quality control specialists, was responsible for monitoring the changes in quality indicators related to STEMI management including DTE time and DTB time as well as implementing appropriate improvement strategies. As a novel quality improvement program, a cardiac triage strategy was introduced in December 2018. Patients who had been diagnosed with STEMI before our ED arrival and/or those who had received resuscitation before ECG acquisition were excluded. All methods were carried out in accordance with relevant guidelines and regulations.

### Pre-intervention management

**Figure 1:**
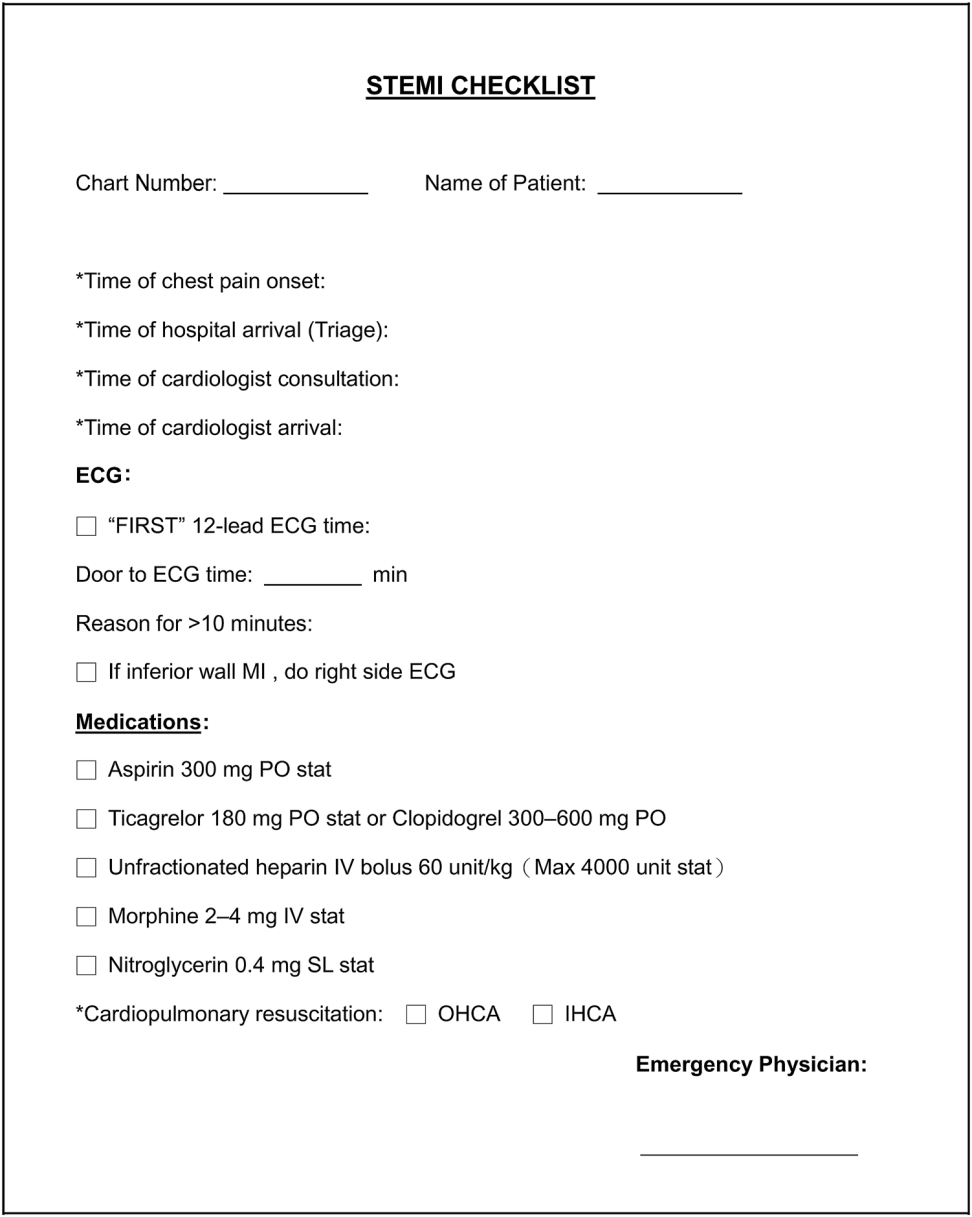
Emergency department checklist for patients with ST-elevation myocardial infarction (STEMI) summarizing timings of key management and medications administered to be completed by emergency physician. *ECG* electrocardiogram, *MI* myocardial infarction, *PO* administration through oral route, *IV* intravenous, *SL* sublingual, *OHCA* out-of-hospital cardiac arrest, *IHCA* in-hospital cardiac arrest.

During the pre-interventional period from November 2017 to November 2018, patients who visited ED were directed to the triage area where they were classified in terms of disease severity and chief complaint into five categories according to the Taiwan Triage and Acuity Scale (TTAS), which was modified from the Canadian Triage and Acuity Scale and has been officially adopted by the Taiwanese emergency health care system since the year 2010: Level I, resuscitation; level II, emergent; level III, urgent; level IV, less urgent; and level V, non-urgent^19^. During this process, patients belonging to level I, II, III, IV, and V should be evaluated by emergency physicians or nurse practitioners immediately, 10 min, 30 min, 60 min, and 120 min, respectively. Because the triage system did not specifically identify patients with potential ischemic cardiac problems, those presenting with typical cardiac symptoms (e.g., chest pain) as well as those having high cardiovascular risks (i.e., age > 50, diagnosis of diabetes) with atypical manifestations (i.e., epigastralgia, nausea, dyspnea, diaphoresis) according to the AHA emergency department screening criteria^20^ had to follow the above management time frame according to the triage classification until the emergency physician identified STEMI and activated the “Code STEMI” to notify cardiologists for evaluation and preparation for PCI. On encountering patients with confirmed STEMI, the emergency physicians were required to complete a DTB checklist summarizing the timing of every step of management (e.g., ECG, consultation with cardiologist) between the time when the patients arrived in ED and the time when they received coronary intervention (Fig. 1). All data were recorded in an electronic database.

### Pilot study period: timing and procedures

**Figure 2:**
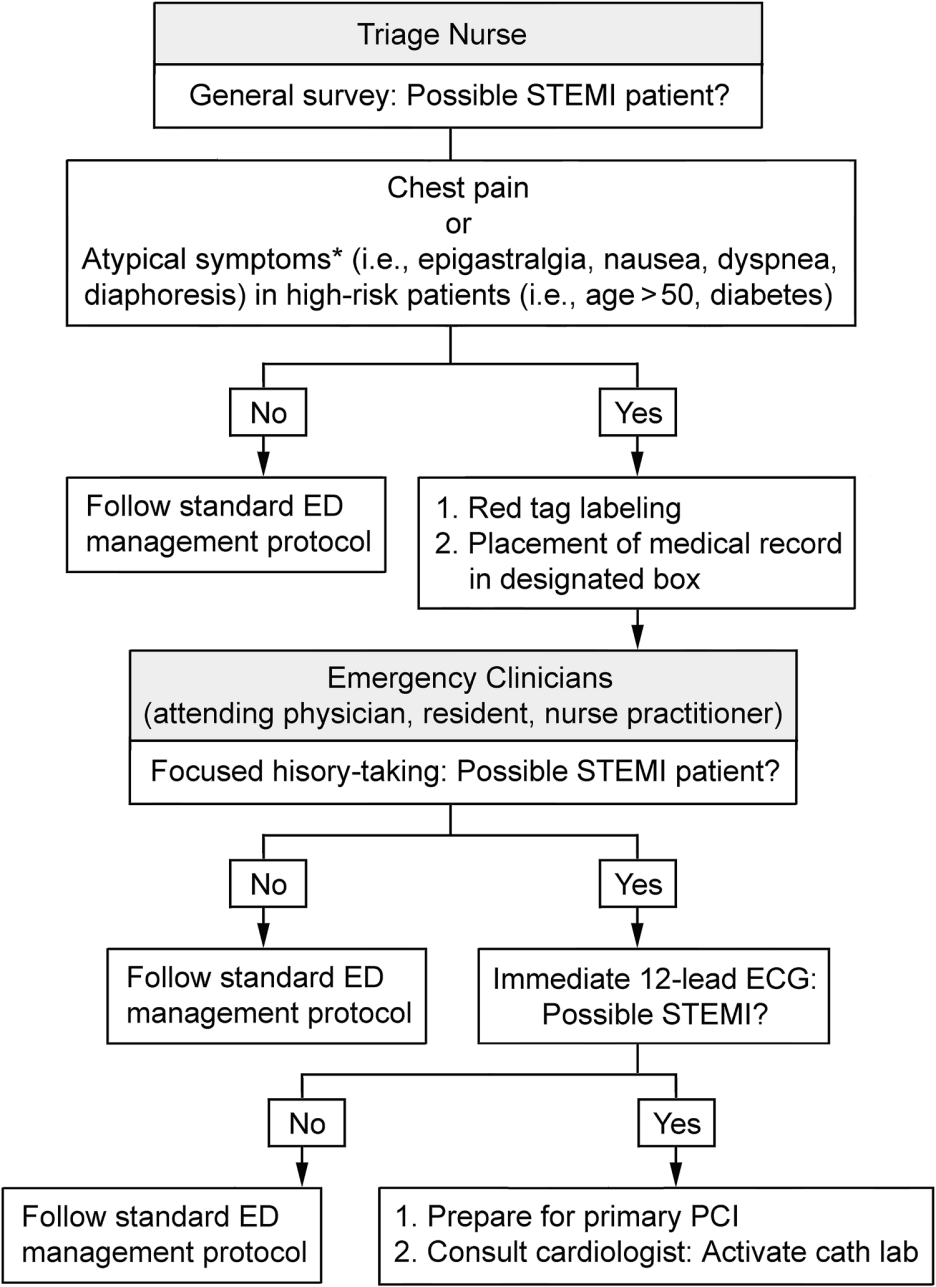
Modified cardiac triage strategy for expediting management of patients with possible ST-elevation myocardial infarction (STEMI). *ED* emergency department, *PCI* percutaneous coronary intervention. *Atypical symptoms according to American Heart Association emergency department screening criteria for STEMI patients. Figure was conducted using Adobe Photoshop version 14 (Adobe Systems Inc., San Jose, CA, https://adobe-photoshop-cc.software.informer.com/14.0/).

In one month’s period (December 2018), in addition to the five-level triage system, a Cardiac Triage program aiming at prioritizing the management of patients with STEMI was introduced in our emergency care system in an attempt to reduce the DTE time. The new strategy involved two key steps to expedite STEMI diagnosis and management (Fig. 2). First, patients who visited ED were directed to the triage area where the triage nurse would immediately identify possible ischemic cardiac symptoms (e.g., chest pain) as well as atypical presentations in those with high cardiovascular risk^20^ and label them with a red tag on bedside or wheelchair and place their medical record in a red designated box next to the seat of ED clinicians. In the meantime, the triage nurse would inform the ED physicians of the patient’s presence. Second, history-taking focusing on the possibility of a STEMI was performed by the emergency physician who ordered immediate 12-lead ECG for patients with suspected STEMI and activated the “Code STEMI” for confirmed cases. All the triage nurses were required to receive on-job training before participating in the cardiac triage program. The algorithm of the cardiac triage protocol (Fig. 2) was also posted in the triage area for their easy reference. At the end of one month, a meeting was held for addressing all issues arising from program implementation to ensure adequate communication and satisfactory problem-shooting between the STEMI quality control team and the frontline emergency physicians and nursing staff. Following confirmation of the feasibility of the program, the new strategy was officially implemented from January 2019.

### Post-intervention follow-up

During the post-intervention period (January 2019 to January 2020), concomitant implementation of the five-class triage system and the Cardiac Triage program had become a routine. Data were continually being recorded electronically on the DTB checklist (Fig. 1) for review. Monthly meetings were held to identify unforeseen problems with patient management and data collection during the process of cardiac triage.

### Outcomes and definitions

Data collection was completed by the end of January 2020 when outcomes of the strategy was assessed by the quality control team through analyzing and comparing the data acquired before and after implementation of the Cardiac Triage program. Data during the intervention period (December 2018) was excluded. The primary outcome of the study was the median DTE time and the achievement rate of DTE time < 10 min, while the secondary outcome was the median DTB time and the achievement rate of DTB time < 90 min as well as in-hospital mortality rate. The achievement rates of DTE time < 10 min and DTB time < 90 min were compared before and after implementation of the cardiac triage program by assessing the changes in the mean achievement rates every three months.

Door time, ECG time and balloon time were defined as the times of registration at ED reception, completion of ECG, and first balloon inflation in culprit lesion, respectively^21^. Daytime and nighttime were defined as the periods from 07:01 to 17:00 and from 17:01 to 07:00, respectively. Weekdays and weekend were defined as from Monday morning to Friday night and from Saturday morning to Sunday night, respectively. For triage categories, high and low triage levels were defined as category I, II and category III, IV, V, respectively.

### Statistical analysis

All data were analyzed by using SPSS version 22 (SPSS Inc, Chicago, IL). Mean values and median values were expressed as mean ± SD and median (IQR) respectively. Student's t-test and Chi-squared test were used for determining the significance of difference among continuous and categorical variables, respectively. Fisher’s exact test was used to identify risk factors related to delayed ECG acquirement (i.e., > 10 min after ED arrival) before and after interventions. Independent sample t-test was used for determining the significance of changes in the achievement rates of DTE time < 10 min and DTB time < 90 min after implementation of the cardiac triage strategy. A two-tailed *p* value of less than 0.05 was considered statistically significant.

### Ethics committee approval

Ethics approval for this study was granted by the Institutional Review Board of the E-DA Hospital (EMRP-109-026) and need for informed consents was waived because of the retrospective nature of the present study.

## Results

### Study population

Between November 2017 and January 2020, there were a total of 136,402 ED visits and 12,058 ECG examinations. Among them, 6,063 visits (8.83%) involved ECG in pre-intervention period and 5995 visits (8.84%) in post-intervention period. Of the 193 patients with the diagnosis of STEMI, 66 had been diagnosed with STEMI before arrival at our ED and the other four had received resuscitation before ECG acquisition. Additionally, after excluding six more patients diagnosed with STEMI during the one-month pilot study period (December 2018), a total of 117 patients were enrolled into this study, including 57 in the pre-intervention group and 60 in the post-intervention group. A review of the characteristics of our patients with STEMI during the study period demonstrated no significant difference in age, gender, triage category, means of ED arrival, visiting time, typical angina presentations, and comorbidities between the pre- and post-intervention groups (Table 1).

**Table 1:** Baseline characteristics of STEMI patients (n = 117).

Characteristics	Pre-intervention group (n = 57)	Post-intervention group (n = 60)	*p* value
Age, y, mean ± SD	62.5 ± 13.9	62.2 ± 11.1	0.91
Male, n (%)	50 (87.7)	52 (86.7)	1.00
^a^**Triage category, n (%)**			0.43
Category 1	13 (22.8)	8 (13.3)	
Category 2	34 (59.6)	42 (70.0)	
Category 3	10 (17.5)	10 (16.7)	
Category 4	0	0	
Category 5	0	0	
**Means of ED arrival, n (%)**			0.12
Walk-in	41 (71.9)	34 (56.7)	
Emergency medical service	16 (28.1)	26 (43.3)	
**ED time, n (%)**			0.14
Daytime	34 (52.6)	27 (60.0)	
Nighttime	23 (47.4)	33 (40.0)	
**ED day, n (%)**			0.46
Weekday	30 (52.6)	36 (60.0)	
Weekend	27 (47.4)	24 (40.0)	
Chest pain, n (%)	49 (86.0)	56 (93.3)	0.39
**Comorbidities, n (%)**			
Diabetes Mellitus	25 (43.9)	24 (40.0)	0.71
Hypertension	31 (54.4)	35 (58.3)	0.71
Dyslipidemia	37 (64.9)	41 (68.3)	0.70
Old cerebrovascular accident	4 (7.0)	4 (6.7)	1.00
Chronic kidney disease	20 (35.1)	25 (41.7)	0.57

*STEMI* ST-elevation myocardial infarction, *ED* emergency department.

^a^Triage categories according to the Taiwan Triage and Acuity Scale (TTAS), a modification of the Canadian Triage and Acuity Scale.

### Primary outcomes after intervention

**Figure 3:**
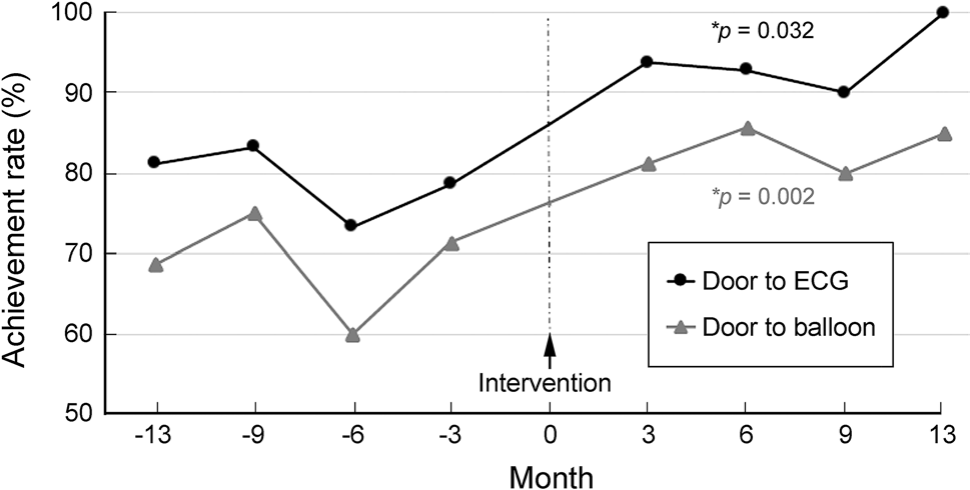
Changes in achievement rates of “Door to ECG” (DTE) time < 10 min and “Door to Balloon” (DTB) time < 90 min after implementation of cardiac triage strategy. *Significance of difference determined by independent sample t-test.

The DTE time was significantly shorter after intervention than that before intervention (4 min vs. 5 min, *p* = 0.02) (Table 2). In addition, there was a higher proportion of patients with DTE < 10 min in the post-intervention group compared with that in the pre-intervention group (95.0% vs. 78.9%, *p* = 0.01, respectively). Comparison of the achievement rate of DTE < 10 min before and after implementation of the cardiac triage program showed a significant trend of increase after initiation of the strategy (*p* = 0.032) (Fig. 3).

**Table 2:** Comparison of outcomes among STEMI patients before and after intervention (n = 117).

Variables	Pre-intervention group (n = 57)	Post-intervention group (n = 60)	*p* value
Door to ECG time (min), median (IQR)	5.0 (1.5–8.0)	4.0 (1.0–5.0)	0.02*
Door to ECG time < 10 min, n (%)	45 (78.9)	57 (95.0)	0.01*
Door to Balloon time (min), median (IQR)	81.0 (70.5–91.5)	70.0 (53.3–84.0)	< 0.01**
Door to Balloon time < 90 min, n (%)	39 (68.4)	50 (83.3)	0.08
Length of stay (day), median (IQR)	6 (5–9)	6 (5–7)	0.73
ICU stay (day), median (IQR)	3 (3–4)	3 (3–4)	0.71
Mortality, n (%)	2 (3.5)	2 (3.3)	1.00

*STEMI* ST-elevation myocardial infarction, *ECG* electrocardiogram, *IQR* interquartile range, *ICU* intensive care unit.

*p < 0.05, **p < 0.01.

### Secondary outcomes after intervention

Consistent with the change in DTE time, the DTB time was significantly shorter after intervention compared to that before intervention (70 min vs. 81 min, *p* < 0.01, respectively) (Table 2). Besides, there was a higher proportion of patients achieving DTB time < 90 min in the post-intervention group than that in the pre-intervention group (83.3% vs. 68.4%, *p* = 0.08, respectively) despite the lack of statistical significance. On the other hand, there was a significant trend of increase in achievement rate of DTB time < 90 min after implementation of the cardiac triage the strategy (*p* = 0.002) (Fig. 3). However, there was no significant difference in the length of hospital stay, intensive care unit (ICU) stay, and in-hospital mortality between the pre- and post-intervention groups.

### Effectiveness of interventions for improving DTE times

Our literature search identified four reported contributors to DTE delays, including STEMI without chest pain, underestimated disease severity (i.e., initially low triage levels of III–V), walk-in patient, and female gender^8–10,15,22^. Therefore, the four factors were used for evaluating the effectiveness of the program for improving DTE times in STEMI patients. There were a total of 15 patients with DTE exceeding 10 min in the present study, including 12 in pre-intervention group and three in post-intervention group. A low triage level was found to be the most significant predictor of outcome improvement because the rate of DTE > 10 min decreased drastically from 90 to 10% after strategy implementation (*p* < 0.01) (Table 3). In addition, the DTE time of walk-in patients was significantly reduced from 29.2 to 8.8% (*p* = 0.04). On the other hand, despite the apparent decrease in the rate of DTE > 10 min in women, it failed to reach statistical significance (*p* = 0.62). In addition, there was no significant impact of STEMI without chest pain on the effectiveness of the cardiac triage program (Table 3).

**Table 3:** Comparison of factors associated with ECG > 10 min between pre- and post-intervention groups.

Reasons for ECG > 10 min	Pre-intervention group ^a^N1/N2 (%)	Post-intervention group ^a^N1/N2 (%)	*p* value
STEMI without chest pain	3/8 (37.5%)	2/4 (50.0%)	1.00
Low triage level	9/10 (90.0%)	1/10 (10.0%)	< 0.01**
Walk-in patient	12/41 (29.2%)	3/34 (08.8%)	0.04*
Female gender	4/7 (57.1%)	3/8 (37.5%)	0.62

*ECG* electrocardiogram, *STEMI* ST-elevation myocardial infarction.

*p < 0.05, **p < 0.01.

^a^N1 represents number of STEMI patients with door-to-ECG > 10 min and N2 represents total numbers of STEMI patients.

## Discussion

The current study demonstrated that implementation of a modified cardiac triage protocol for early identification and treatment of patients with STEMI in the emergency care setting could significantly shorten the median DTE time and increase the achievement rate of DTE time less than 10 min. Moreover, median DTB time was also significantly reduced. Further investigation also revealed a significant reduction in the incidence of DTE time over 10 min among patients belonging to a low triage category (i.e., III, IV, or V) after intervention.

DTB is a survival chain comprising early ECG with prompt interpretation, early catheterization lab activation, an expedited response to activation, and rapid reperfusion^17^. Although multiple factors would affect DTB time, a previous study has shown a stronger association of DTB time with door-to-activation time compared to that with activation-to-laboratory and laboratory-to-balloon times^5^. Timely ECG is crucial to the identification of patients with STEMI for prompt primary PCI. The American Heart Association (ACC/AHA) management guideline for patients presenting with symptoms of cardiac ischemia has indicated a DTB time of less than 10 min as a standard for acceptable emergency medical practice^1^. Hence, various efforts have been made to shorten the DTE time, including designation of an ECG technician and equipment for triage ECG, organization of triage education, improvement of triage disposition, and data feedback^13^. Although assigning a technician and ECG equipment to the conduction of triage ECG has been shown effective for reducing DTE time^8,17,23–25^, indiscriminate ECG screening without a patient interview by an experienced emergency physician has raised the concern of increasing workload among nursing staff as well as the possibility of low cost-effectiveness^16^. Indeed, a previous study has reported a 30% increase in ECG workload after implementation of a triage ECG program^10^. By combining the strategies of cardiac triage and triage ECG, Coyne et al. have shown a reduction of DTE time by 39% (i.e., from 23 to 14 min) and DTB by 12% (from 85 to 75 min). Taking into consideration the downsides of triage ECG, the current study aims at investigating the impact of cardiac triage per se on DTE time reduction.

Our cardiac triage protocol included the triage nurse’s early identification of patients with a possible ischemic heart disease by labeling the patients a red warning tag that alerted the emergency medical personnel (i.e., emergency physicians, residents, or nurse practitioners) of the need for prompt history-taking and placing their medical records in a designated box for expedited management. For patients presenting with a history suggestive of coronary heart disease, prompt ECG was acquired. In this way, indiscriminate ECG screening was avoided. This approach also eliminated the necessity of assigning nursing staff, space, and ECG equipment as required for triage ECG. This is of particular clinical importance because overcrowding in the ED is a critical issue worldwide^26,27^ and efficient utilization of medical manpower remains one of the formidable challenges to healthcare organizations. Moreover, although the proportion of patients (8.84%) receiving ECG in our ED in the post-intervention group was not increased compared with that in the pre-intervention group (8.83%), our study demonstrated that the achievement rate of DTE < 10 min and DTB < 90 min were improved from 78.9 to 95% (20.4%) and 68.4 to 83.3% (21.8%), respectively (both *p* < 0.05). The findings, therefore, indicate significant reductions in both DTE and DTB without increasing the ECG workload.

Furthermore, through adopting the concept of mass casualty triage^28^, the triage nurse labeled the patients suspected of experiencing acute coronary syndrome with a red tag and placed their medical records in a designated box to expedite medical attention by emergency clinicians in a busy and noisy environment as well as the acquisition of an ECG for early diagnosis, thereby enabling prompt primary PCI for confirmed cases of STEMI.

As a DTE time over 10 min is an indicator of unacceptable emergency medical practice^1^, we investigated the effectiveness of our interventions for reducing the DTE time by selecting the predictors previously reported to be related to DTE > 10 min, including the female gender^9^, STEMI without chest pain^10,15^, relatively non-severe initial presentations (i.e., Triage Category III, IV, V)^22^, and walk-in patients^8^, for analysis. Among them, DTE time of STEMI patients assigned into a low (i.e., less severe) triage category and walk-in patients were significantly reduced after intervention. The designation of triage levels to patients with cardiac ischemic symptoms by triage nurses might be affect by multiple factors, including patient’s characteristics, acute myocardial infarction volume, or subjective experience of triage nurses^22,29^. Clare et al. has reported that up to one third of patients with STEMI could have an initial non-severe presentation (i.e., a low triage score), resulting in prolonged DTE and DTB times^29^. Albeit not as high as the proportion previously reported, there were still 17% of STEMI patients being assigned to a low triage category in our study. Although there was no significant difference in the proportion of patients with a low triage score between pre- and post-intervention groups in the current study as well as in a previous triage ECG report^22^, the percentage of patients with STEMI assigned with a low triage score decreased significantly from 90% (9 of 10) to 10% (1 of 10) (*p* < 0.01) after cardiac triage implementation. Additionally, the mode of arrival may also contribute to a prolonged DTE time^30^. Literature review showed that patients with walk-in arrival are more likely to be designated into a low triage category compared with those arrived by ambulance^31^, contributing to a possible delay in receiving medical attention under the circumstances of ED overcrowding. This is supported by our study in which all STEMI patients with DTE > 10 min arrived at the ED on foot in both pre-and post-intervention periods. Utilizing cardiac triage with a red warning tag could expedite ECG examination for patients presenting with ischemic cardiac symptoms even if they belong to a low triage category.

Despite the lack of statistical significance, DTE > 10 min in the female gender was decreased after our intervention. Female gender has been reported as a strong independent predictor of delayed ECG acquisition in several literature reviews^12,22,32^. Possible reasons for delayed ECG in females include atypical symptom presentation and the concern for ECG acquisition-related violation of personal privacy to which a sufficient number of female triage nurses has been reported to be a possible solution^9^. During the post-intervention period, all female patients undergoing cardiac triage received ECG performed by female nurse practitioners so that the influence of personal privacy on DTE time could be minimized.

There were 14% and 6% of STEMI patients without chest pain in our pre- and post-invention groups, respectively. The figure was within the range of 6–9% previously reported^15,33^. Our results showed no significant difference in the rate of DTE time < 10 min before and after implementation of the cardiac triage program (37.5% vs. 50%, respectively), indicating no notable benefit in this particular patient population. One of the possible reasons could be atypical initial presentations of STEMI such as general discomfort, dizziness or weakness in some of the patients, which have not been included in the AHA screening guidelines^20^. Further emendations of the cardiac triage protocol may be necessary to expand the criteria for inclusion. Nevertheless, the number of patients with atypical STEMI presentations was too small to arrive at a robust conclusion.

The present study had its limitations. Firstly, the statistical power and reliability of our results were limited by the relatively small number of patients, which was due to the single center nature of the current study instead of a nationwide investigation. Besides, STEMI patients usually comprise only a minor portion of patients visiting the ED during the study period. Second, because the modified cardiac triage protocol is aimed at expediting STEMI patient management in a high-volume emergency care setting as a quality improvement strategy, its feasibility and effectiveness in other ED settings remain to be validated. Third, the accuracy of data acquisition may be hampered by ambiguous symptom descriptions in medical records, for which experts in the quality control team were recruited as reviewers to categorize the nature of those symptoms to minimize the impact of this potential confounder.

In conclusion, utilizing a modified chief complaint-based cardiac triage strategy, the current study showed that the DTE and DTB times could both be significantly shortened for STEMI patients. Moreover, the pitfall of failure in early discrimination of patients with STEMI associated with the conventional triage system could also be improved as reflected by the shortened ECG time in STEMI patients with initially underestimated disease severity.

## Data Availability

Data relevant to the present study are available on request made to the corresponding author.
